# Super-selective robot-assisted partial nephrectomy using near-infrared flurorescence versus early-unclamping of the renal artery: results of a prospective matched-pair analysis

**DOI:** 10.1590/S1677-5538.IBJU.2017.0311

**Published:** 2018

**Authors:** Cecilia Lanchon, Valentin Arnoux, Gaëlle Fiard, Jean-Luc Descotes, Jean-Jacques Rambeaud, Jean-Benjamin Lefrancq, Delphine Poncet, Nicolas Terrier, Camille Overs, Quentin Franquet, Jean-Alexandre Long

**Affiliations:** 1 Department of Urology, Grenoble University Hospital, Grenoble, France;; 2 UJF-Grenoble 1, CNRS, INSERM, TIMC-IMAG UMR 5525, 38041 Grenoble, France

**Keywords:** Nephrectomy, Kidney, Ischemia

## Abstract

**Introduction:**

Super-selective clamping of tumor-specific segmental arteries was developed to eliminate ischemia of the remnant kidney while limiting hemorrhage during partial nephrectomy. The objective is to evaluate the benefice of super-selective clamping on renal functional outcome, compared to early-unclamping of the renal artery.

**Materials and Methods:**

From March 2015 to July 2016, data from 30 patients undergoing super-selective robot-assisted PN (RAPN) for a solitary tumor by a single surgeon were prospectively collected. Tumor devascularization was assessed using indocyanine green near-infrared fluorescence. A matched-pair analysis with a retrospective cohort undergoing early-unclamping was conducted, adjusting on tumor complexity and preoperative eGFR. Perioperative, oncologic and functional outcomes using DMSA-renal scintigraphy were assessed. Multivariate analysis was performed to identify predictors of postoperative renal function and *de novo* chronic kidney disease (CKD).

**Results:**

Super-selective RAPN was successful in 23/30 patients (76.7%), 5 requiring secondary main artery clamping due to persistent tumor fluorescence. Matched-pair analysis showed similar operating time, blood loss, positives margins and complication rates. Super-selective clamping was associated with an improved eGFR variation at discharge (*p*=0.002), 1-month (*p*=0.01) and 6-month post-op (-2%vs-16% *p*=0.001). It also led to a better relative function on scintigraphy (46%vs40% *p*=0.04) and homolateral eGFR (*p*=0.04), and fewer upstaging to CKD stage ≥3 (*p*=0.03). On multivariate analysis, super-selective clamping was a predictor of postoperative renal function.

**Conclusion:**

Super-selective RAPN leads to an improved preservation of renal function and a reduced risk of *de novo* CKD stage≥3, while keeping the benefit of main artery clamping on perioperative outcomes.

## INTRODUCTION

Optimizing functional outcome has become one of the foremost challenges in localized renal cancer treatment. Duration of ischemia remains a major surgically modifiable component during partial nephrectomy (PN). Although a warm-ischemia time (WIT) ≤25 minutes seems commonly accepted, depriving the whole kidney from arterial blood flow may lead to ischemic injuries on healthy parenchyma. Therefore, superselective clamping of segmental arteries directly feeding the tumor has emerged to limit ischemia to the targeted tumor area.

The objective of this matched-pair study is to assess the benefit of super-selective robot-assisted partial nephrectomy (RAPN) on postoperative renal function preservation, compared to conventional RAPN with early-unclamping.

## MATERIALS AND METHODS

### Study population

#### Zero-ischemia RAPN group

From March 2015 to July 2016, patients suitable for super-selective (“zero-ischemia”) RAPN using indocyanine green (ICG) near-infrared fluorescence (NIRF) for a solitary renal tumor were prospectively included. Approval from our institutional ethics committee was obtained (CECIC Rhône-Alpes-Auvergne, Grenoble, IRB5891). All surgeries were carried out by a single experienced surgeon, who had performed over 200 laparoscopic and robotic procedures.

All patients >18 diagnosed with a single renal tumor, candidates for a RAPN with superselective clamping on preoperative imaging were enrolled.

Patients presenting solitary kidney and any medical history forbidding RAPN were excluded. Patients requiring per-operatively a main artery clamping were secondarily excluded from the analysis.

#### Control group: early-unclamping

Successful cases of zero-ischemia RAPN were matched 1:1 with a control group of patients who underwent RAPN with early-unclamping of the renal artery performed by the same surgeon before the inclusion period. Matched-pair analysis was performed using propensity score matching, calculated to adjust for RENAL score and preoperative estimated glomerular filtration rate (eGFR).

### Data collection

Demographic data, tumor characteristics, preoperative renal function (serum creatinine and eGFR measured with the CDK-EPI equation), operative parameters (WIT, operative time and blood loss), post-operative outcome (length of stay, pathological surgical margins), post-operative complications using the Clavien-Dindo classification, and renal function at discharge, 1 and 6-month follow-up were collected.

Preoperative planning required cross-sectional imaging with computed tomography (CT), which included arterial and venous phases with 3D-reconstructions to visualize tumor feeding vessels, proximity of the renal sinus and collecting system. RENAL, PADUA nephrometry and Mayo Adhesive Probability (MAP) scores were calculated. Dimercapto-succinic acid (DMSA) renal scintigraphy was performed at 6-month follow-up to assess split renal function.

### Surgical technique

RAPN was performed with a DaVinci Si^®^ system using a transperitoneal approach. The surgical technique for zero-ischemia RAPN has been described elsewhere (1, 2), consisting in a vascular microdissection of the renal hilum to control tertiary or higher-order renal artery branches directly feeding the tumor. Preoperative knowledge of arterial anatomy, through a careful study of the 3D CT-scan images, was mandatory to properly identify target arteries. Scanlan^®^-Bulldog Clamps were applied to achieve tumor-specific devascularization. NIRF was then performed using an intravenous injection of 0.5 to 2cc of ICG to confirm both tumor devascularization and ongoing perfusion of the remaining healthy parenchyma before starting tumor excision. Indeed, when selective clamping was efficient, the tumor remained dark while the rest of the kidney appeared fluorescent green, allowing for real-time verification that the appropriate arterial blanches were controlled (Figure-1). On the contrary, a persistent fluorescent uptake of the tumor and peri-tumor area after selective clamping of the targeted arteries indicated a persistent blood flow alerting of the high risk of hemorrhage during tumor resection. Safety then dictated to convert to main artery clamping. Tumor excision, whether under super-selective or conventional clamping, was carried out with a combination of electrocautery and cold scissors. According to the initial technique proposed by Baumert (3) and Nguyen (4), a running suture using 3/0 V-loc™ is performed to provide hemostasis of the tumor bed. Then clamps are removed allowing to decrease ischemia time. Renorraphy was then achieved using a 0 Vicryl™ suture secured with Hem-o-lok^®^ clips.

### Statistical analysis

Statistical analysis was obtained with SPSS^®^, version 21. Propensity score matching was conducted using the PS matching custom dialog for SPSS. In a first step, propensity score was estimated using logistic regression. Matching was then performed using a 1:1 nearest neighbor matching. Continuous variables were reported using median values with interquartile ranges (IQR) and compared with the independent Mann-Whitney *U*-test. Categorical variables were reported as counts and proportions (%) and compared using the χ^2^ test or Fisher exact test as appropriate.

Multiple linear regression was performed to identify predictors of postoperative renal function (i.e. %variation in serum creatinine and eGFR at 6 months), as well as postoperative split renal function. Variables attaining *p*≤0.05 on univariate analysis or considered clinically relevant were included in the multivariate analysis.

Multiple logistic regression was used to study predictors of *de novo* renal chronic kidney disease (CKD) on follow-up.

## RESULTS

In the study period, zero-ischemia RAPN for a solitary tumor was attempted in 30 patients. Among them, 23 patients (76.7%) had successful super-selective tumor devascularization confirmed by NIRF. Persistent perfusion in the tumor area was revealed on NIRF in 5 cases for which main artery clamping was required. NIRF revealed slightly fluorescent tumor edges in 2 patients, for whom zero-ischemia RAPN was attempted anyway. Although both procedures were successful without the need for main artery clamping, parenchymal bleeding during tumor excision led to a substantial blood loss in each case (700cc and 1000cc). None of them experienced postoperative complications. One of these patients required transfusion.

### Demographics and immediate postoperative outcome

Matching was successful for all 25 cases, as shown by the equivalent baseline and tumors characteristics between zero-ischemia and early-unclamping patients (Table-1). Operative parameters and immediate results were similar in both cohorts in terms of operative time, WIT, blood loss, transfusion rate and hospital stay (Table-2). Overall final pathology revealed malignant tumors in 74% of cases, with a majority of clear-cell carcinoma (48%). A positive surgical margin occurred in 1 patient (4%) in each group. No adverse reaction to ICG was noted.

**Table 1:** Demographics and preoperative characteristics in the zero-ischemia and early-unclamping cohorts.

		Total n=50	Zero-ischemia n=25	Early-unclamping n=25	*p* value
Age, yrs		67 (57-72)	66 (59-73)	68 (56-72)	0.92
Male gender		38 (76)	22 (88)	17 (68)	0.19
Charlson score		5 (4-6)	5 (4-6)	5 (4-7)	0.94
BMI		26 (23-28)	27 (23-28)	25 (23-29)	0.90
Left side		20 (40)	8 (32)	11 (44)	0.38
Solitary kidney		1 (2)	1 (4)	0 (0)	0.31
Previous biopsy		5 (10)	1 (4)	4 (16)	0.16
Cystic tumor		8 (16)	3 (12)	5 (20)	0.44
Tumor size on CT, cm		33 (30-42)	30 (27-35)	40 (30-45)	0.07
**RENAL score**					**0.78**
	4-6	12 (24)	7 (28)	5 (20)	
	7-9	24 (48)	11 (44)	13 (52)	
	10-12	14 (28)	7 (28)	7 (28)	
**PADUA Score**					**0.41**
	6-7	11 (22)	7 (28)	4 (16)	
	8-9	24 (48)	11 (44)	13 (52)	
	≥10	15 (30)	7 (28)	8 (32)	
**MAP score**					**0.21**
	0-3	37 (74)	20 (80)	17 (68)	
	4-5	13 (26)	5 (20)	8 (32)	
Hemoglobin, g/dL		14.5 (13-15)	14.5 (13.1-16)	14.1 (12.6-15)	0.17
Serum creatinine, μmol/L		86 (76-96)	92 (77-101)	83 (71-97)	0.34
eGFR, mL/min		74 (66-89)	75 (65-89)	73 (66-92)	0.82
**CKD-EPI stage**					**0.18**
	1	9 (18)	2 (8)	7 (28)	
	2	32 (64)	18 (72)	14 (56)	
	3	9 (18)	5 (20)	4 (16)	

**BMI =** Body Mass Index; **CT =** Computed Tomography; **eGFR =** estimated Glomerular Filtration Rate (CKD-EPI formula); **MAP score =** Mayo Adhesive Probability score; **IQR =** interquartile ranges Data are shown as median (IQR) or number (percentage)

**Table 2:** Comparison of operative and postoperative parameters.

		Total n=50	Zero-ischemia n=25	Early unclamping n=25	*p* value
Clamping time, min		15 (11-19)	14 (10-18) [selective clamping]	15 (13-19)	0.12
Operating time, min		120 (90-150)	123 (90-143)	119 (95-150)	0.51
Blood loss, mL		101 (50-300)	105 (50-238)	95 (50-300)	0.74
Toxic peri-nephric fat		15 (30)	9 (36)	6 (24)	0.36
Length of stay, d		5 (4-6)	5 (4-6)	5 (5-6)	0.59
Hemoglobin at discharge, g/dL		117 (106-130)	117 (106-128)	117 (106-132)	0.75
Transfusion		2 (4)	1 (4)	1 (4)	0.98
**Histology**					**0.33**
	10 (20)	5 (20)	5 (20)		
	2 (4)	2 (8)	0 (0)		
	8 (16)	4 (16)	4 (16)		
	2 (4)	1 (4)	1 (4)		
	3 (6)	0 (0)	3 (12)		
	2 (4)	1 (4)	1 (4)	0.98	
				**0.71**	
	9 (18)	5 (20)	4 (16)		
	0 (0)	0 (0)	0 (0)		
	Clavien III-IV				

**eGFR =** estimated Glomerular Filtration Rate (CKD-EPI formula)**; IQR =** Interquartile ranges Data are shown as median (IQR) or number (percentage)

### Complications

Early adverse events (within 30 days of surgery) were similar in both groups, with no major complications occurring during the study period (Table-2). Patients undergoing zero-ischemia RAPN suffered 5 (20%) minor complications, including 1 unexplained fever, 2 infections (1 respiratory and 1 urinary), 1 regressive acute renal failure and 1 parietal hematoma. They were equivalent to those observed in the early-unclamping group (1 unexplained fever, 2 urinary tract infections and 1 regressive acute renal failure, *p*=0.71).

### Renal functional outcome

Assessment of renal function was available for all patients at discharge, 1 and 6 months postoperatively.

In the present study, WIT was longer than 20 min in 5 cases (20%) in the early unclamping group vs 3 (12%) in the zero-ischemia group. The latter may have, however, a less pronounced impact since devascularisation is limited.

Compared to early-unclamping, zero-ischemia RAPN was associated to more favorable renal functional outcomes, as shown by the lower percent change in serum creatinine and eGFR at discharge (*p*=0.002), 1-month (*p*=0.01) and 6-month follow-up (-2% vs -16% *p*=0.001, Figure-2). Upstaging to CKD stage ≥3 within 3 months was significantly reduced after zero-ischemia RAPN (4% vs 24% *p*=0.03, Table-3). Functional evaluation of the operated kidney was possible for all patients but 2 who did not undergo DMSA renal scintigraphy due to a history of contralateral PN (1 in each group). Split renal function of operated kidney was superior after super-selective RAPN compared to main artery clamping both on DMSA scintigraphy (46% v s40% *p*=0.04, Figure-3) and homolateral eGFR (34 v s27μmol/L *p*=0.04).

**Table 3:** Evaluation of postoperative renal function after zero-ischemia or early-unclamping RAPN.

		Total	Zero-ischemia	Early unclamping	*p* value
**Serum creatinine, μmol/L**					
	Discharge	90 (67-105)	88 (71-103)	95 (82-110)	0.26
	M1	91 (75-112)	90 (76-110)	93 (74-116)	0.69
	M3	90 (78-106)	88 (76-101)	95 (80-107)	0.40
**% Variation in creatinine**					
	preop - discharge	+8% (-9; +23)	-1% (-11; +8)	+14% (+1; +29)	0.003*
	preop - M1	+10% (0; +21)	+1% (-1; +17)	+15% (+4; +31)	0.02*
	preop - M3	+10% (0; +20)	+3% (-1; +9)	+17% (+10; +22)	0.001*
**eGFR, mL/min**					
	Discharge	73 (59-87)	75 (64-95)	70 (54-85)	0.31
	M1	71 (55-87)	74 (60-88)	69 (53-87)	0.30
	M3	71 (56-85)	73 (66-89)	66 (53-83)	0.17
**% Variation in eGFR**					
	preop - discharge	-6% (-18; 0)	0 (-9; +12)	-15% (-24; +1)	0.002*
	preop - M1	-8% (-18; +9)	-1% (-13; +5)	-13% (-22; -4)	0.01*
	preop - M3	-9% (-17; 0)	-2% (-9; +3)	-16% (-19; -11)	0.001*
**Renal function of operated kidney**					
	DMSA scintigraphy, %	44 (37-48)	46 (41-49)	40 (36-45)	0.04*
	Split eGFR, mL/min	31 (23-37)	34 (26-41)	27 (22-34)	0.04*
	De novo CKD stage ≥ 3	7 (14)	1 (4)	6 (24)	0.03*

**CDK** = Chronic Kidney Disease; **DMsA** = Dimercapto-succinic acid; **eGFR** = estimated Glomerular Filtration Rate (CKD-EPI formula); **IQR** = Interquartile ranges; **RAPN** = Robot-Assisted Partial Nephrectomy Data are shown as median (IQR) or number (percentage)

### Predictors of postoperative renal function and de novo chronic kidney disease

On multivariate analysis, when adjusted to Charlson score, initial renal function (eGFR or serum creatinine), RENAL nephrometry score and WIT, type of ischemia (super-selective or conventional) was the only predictor of percent variation in eGFR and serum creatinine at 6 months (β=0.41 *p*=0.01 and β=-0.38 *p*=0.02 respectively, Table-4).

**Table 4:** Predictors of 3-month percent variation in serum creatinine (μmol/L), eGFR (mL/min), DMSA renal scintigraphy (%) and split eGFR (μmol/L) on multivariate analysis.

	Variable	β	IC 95%	*p* value
**Serum creatinine variation**	Preoperative creatinine	- 0.12	- 0.004; 0.001	0.20
	Charlson score	0.01	- 0.02; 0.03	0.97
	RENAL score	0.08	- 0.05; 0.09	0.61
	WIT	0.03	- 0.01; 0.01	0.86
	Surgical technique	- 0.38	- 0.08; 0.58	0.02*
**Global eGFR variation**	Preoperative eGFR	- 0.16	- 0.01; 0.01	0.35
	Charlson score	0.04	- 0.02; 0.03	0.78
	RENAL score	- 0.12	- 0.11; 0.05	0.44
	WIT	0.14	- 0.01; 0.02	0.41
	Surgical technique	0.41	0.03; 0.26	0.01*
**DMSA scintigraphy**	Preoperative eGFR	- 0.06	-0.27; 0.18	0.73
	Charlson score	0.17	- 0.90; 2.86	0.30
	RENAL score	- 0.32	- 9.87; 0.09	0.05*
	WIT	0.29	- 0.10; 1.28	0.09
	Surgical technique	0.35	- 0.70; 14.66	0.03*
**Split eGFR variation**	Preoperative eGFR	0.36	- 0.04; 0.54	0.02*
	Charlson score	0.09	- 1.46; 2.72	0.55
	RENAL score	- 0.24	- 9.80; 1.29	0.13
	WIT	0.26	- 0.17; 1.37	0.13
	Surgical technique	0.43	3.01; 18.54	0.008*

**DMsA =** Dimercapto-succinic acid; **eGFR =** estimated Glomerular Filtration Rate (CKD-EPI formula); **WIT =** Warm Ischemia Time

Concerning the evaluation of operated kidney, ischemia type and RENAL score were predictors of 6-month renal function on DMSA scintigraphy (β=0.35 *p*=0.03 and β=-0.32 *p*=0.05 respectively, Table-4). Zero-ischemia and initial eGFR were predictors of homolateral split eGFR.

Finally, no independent predictors of *de novo* CKD stage ≥3 were identified, although a trend was noted in favor of super-selective clamping (OR=0.11 *p*=0.06).

## DISCUSSION

We herein report the results of a matched-pair analysis comparing renal functional outcome of zero-ischemia RAPN using indocyanine green fluorescence to main artery early-unclamping. Over time PN surgery has sustained several technical refinements with the overall goal to reach an ideal PN, which would achieve the best possible functional outcome, while ensuring oncological safety. Early-unclamping emerged with the expansion of laparoscopic PN to reduce ischemia time, as it resulted in a 10-min longer WIT compared to open PN (5). Initial descriptions report a 50% reduction of WIT due to early-unclamping (3,4), with a trend towards a reduction of 3-month postoperative complications, and eGFR decline (4). Early-unclamping in RAPN, showed the same benefice on WIT compared to conventional clamping (6). However, depriving the whole kidney from arterial blood flow during tumor excision may lead to ischemic injuries on healthy parenchyma (7, 8).

### The concept and safety of zero-ischemia PN

In 2010, Gill described the concept of “*zero-ischemia PN",* based on an anatomic microdissection of tumor-specific segmental arteries, up to the tertiary or higher-order branches (1), in order to eliminate ischemia of the remnant kidney while assuring a satisfying hemostasis during tumor excision. In our study, zero-ischemia RAPN was successful in 23 out of 30 patients (76.7%), provided an accurate preoperative planning through segmental imaging. All patients were matched based on tumor complexity and preoperative eGFR in order to account for the other main parameters potentially influencing functional outcome. Perioperative outcome showed no difference in terms of operative times, blood loss, transfusion rate or positive margins, reflecting the feasibility and safety of zero-ischemia, both on a clinical and oncological level. Indeed, only minor complications occurred, at an equivalent rate for both techniques. Several international teams report the feasibility and safety of zero-ischemia PN, with a benefit in terms of functional outcome (9-12), although some found slightly longer operative times (9, 12) or blood loss (9).

### The renal functional benefice of zero-ischemia PN

The main goal behind zero-ischemia PN is an improvement of postoperative renal function due to the persistent blood flow in healthy parenchyma during tumor excision. The present study supports this theory, as variation in eGFR and serum creatinine favored zero-ischemia RAPN on all follow-up measurements. Super-selective clamping also led to fewer upstaging to CKD stage ≥3. Even more, super-selective clamping was the only predictor of 6-month postoperative renal function, when adjusted to other factors potentially influencing functional outcome, such as tumor characteristics and WIT. These findings are consistent with other recent reports. The same benefit on eGFR variation at discharge was supported by Harke, McClintock, Boroskfy (-6.2% vs -18.6% *p*=0.045; -1.9% vs -16.8% *p*<0.01 and -1.8% vs -14.9% *p*=0.03 respectively) (9-11). Despite larger and more complex tumors in the zero-ischemia group, Desai found a lower eGFR decrease at discharge and 4 to 6-month follow-up (11% vs 17% *p*=0.03) (12). Shao, in a series of 75 patients, also reported the benefit of super-selective clamping on 3-month eGFR variation (13). More interestingly, when comparing patients requiring secondary main artery clamping due to an unsuccessful super-selective procedure, eGFR variation was equivalent to the initial conventional PN group (-25.5% vs -26.2% *p*=0.65), attesting for the likely protective role of segmental clamping.

However, most authors only assess global renal function, without taking under account the potential compensation of contralateral kidney, thus undermining the impact on the injured one. The strength of our study was to evaluate the impact of super-selective versus global ischemia directly on operated kidney. DMSA-scintigraphy showed a significantly higher relative renal function in case of super-selective clamping (46% vs 40% *p*=0.04), leading to a higher homolateral eGFR. Despite the absence of preoperative DMSA--scintigraphy, the superior outcome of injured kidney further shows the benefit of zero-ischemia on renal function preservation.

### The contribution of ICG near-infrared fluorescence

Zero-ischemia PN has certain requirements to be successful, among which a meticulous preoperative planning using 3-dimensional CT angiography to assess arterial anatomy. It also requires the ability to verify intraoperatively the absence of tumor vascularization before excision to ensure adequate hemostasis. Several methods have been reported, such as Doppler ultrasound (1, 12, 14), or NIRF using ICG intravenous injections (9-12). In our study, we used NIRF imaging to confirm tumor devascularization, as it provides an easy and quick visualization of the quality of super-selective clamping. Only 30 seconds after indocyanine injection and a switch between white-light to Firefly mode on the Da Vinci console, the surgeon can verify tumor extinction before excision. Doppler ultrasonography, although providing similar information, requires a specific set-up and is, as always, operator-dependant. On the contrary, NIRF can easily be used by any operator, and is all-the-more valuable during the learning curve period. In case of a persistent perfusion, the peri-tumor area will appear fluorescent green immediately warning the surgeon of an incomplete tumor clamping and a potentially increased bleeding risk. In the present study, 5/30 patients (16.7%) underwent secondary main artery clamping due to persistent fluorescence, which is consistent with current literature (9). Two patients underwent zero-ischemia PN despite slightly fluorescent edges, which led to significant bleeding during tumor excision, suggesting the potentially valuable assistance of NIRF. Although unsuccessful cases did not reveal any specific tumor characteristics, several reasons were identified. First, preoperative imaging must include an accurate angiogram to properly assess renal vasculature. To do so, either a quality partnership with radiologists who clearly understand the surgery's requirements or a self-imaging training is mandatory. Nonetheless, even with a preoperative identification of feeding arteries, deviation from the initial direction of the segmental artery during dissection may cause inappropriate clamping. Finally, per-operative bleeding from parenchymal defect may occur, resulting in a choice between main artery clamping or an increased blood loss.

### Zero-ischemia PN: is it relevant for all renal tumors?

While this study was conducted including all tumors regardless of location, zero-ischemia PN appears best suited for medially located or hilar tumors for which vascularization can be meticulously evaluated on preoperative imaging and target arteries can be progressively dissected up to distally located supplying branches (1, 2, 12). Conversely, lateral masses are more likely supplied by multidirectional intrarenal branches, making them less opportune to super-selective vascular control (15). Due to the superficial visualization of ICG on renal cortex, we believe that ICG has limitations to assess deep devascularization. It emphasizes that an accurate preoperative CT evaluation is mandatory to ensure that deep parenchyma is adequately controlled.

### Limitations

The first limitations are the limited cohort and retrospective nature of matched-pair studies, even though follow-up in the zero-ischemia group was prospective. Patient's matching on nephrometry score and preoperative eGFR was done to account for the potential selection bias inherent to the absence of randomization. The main shortcoming was the missing assessment of CT-volumetric parameters, and their potential influence on functional outcome. Indeed, if it is admitted that the amount of spared parenchyma is correlated to postoperative renal function (7, 16), there is an ongoing debate over which of ischemia time or volume preservation has the most impact on postoperative renal function. Nonetheless, on multivariate analysis, taking under account RENAL score, which is a reflection of tumor size and depth, therefore indirectly of volume preservation, super-selective clamping was the only predictor of postoperative eGFR. Furthermore, recent reports suggest that super-selective PN surgery may actually lead to a greater volume preservation compared to conventional clamping (12).

Although not significant, tumor size was slightly smaller in the early unclamping group than in the zero-ischemia group.

Finally, given the retrospective nature of the control group, preoperative DMSA renal scans were not available.

## CONCLUSIONS

The concept of zero-ischemia RAPN emerged from the endless desire for technical refinement to achieve the best possible functional outcome for patients. Our study showed super-selective clamping with NIRF using ICG to be safe and feasible, leading to an increased preservation of overall and split postoperative renal function, while keeping the benefit of main artery clamping on blood loss and perioperative complications.

## ETHICAL APPROVAL

All procedures performed in studies involving human participants were in accordance with the ethical standards of the institutional and/or national research committee and with the 1964 Helsinki declaration and its later amendments or comparable ethical standards.

## INFORMED CONSENT

Informed consent was obtained from all individual participants included in the study.
